# Structural features of T-DNA that induce transcriptional gene silencing during agroinfiltration

**DOI:** 10.5511/plantbiotechnology.23.0719a

**Published:** 2023-12-25

**Authors:** Emi Iida, Kazunori Kuriyama, Midori Tabara, Atsushi Takeda, Nobuhiro Suzuki, Hiromitsu Moriyama, Toshiyuki Fukuhara

**Affiliations:** 1 Department of Applied Biological Sciences, Tokyo University of Agriculture and Technology, Fuchu, Tokyo 183-8509, Japan; 2 Institute of Global Innovation Research, Tokyo University of Agriculture and Technology, Fuchu, Tokyo 183-8509, Japan; 3 Ritsumeikan-Global Innovation Research Organization, Ritsumeikan University, Kusatsu, Shiga 525-8577, Japan; 4 College of Life Sciences, Ritsumeikan University, Kusatsu, Shiga 525-8577, Japan; 5 Institute of Plant Science and Resources, Okayama University, Kurashiki, Okayama 710-0046, Japan

**Keywords:** agroinfiltration, *Nicotiana benthamiana*, small interfering RNA, T-DNA, transcriptional gene silencing

## Abstract

*Agrobacterium tumefaciens* (*Rhizobium radiobacter*) is used for the transient expression of foreign genes by the agroinfiltration method, but the introduction of foreign genes often induces transcriptional and/or post-transcriptional gene silencing (TGS and/or PTGS). In this study, we characterized the structural features of T-DNA that induce TGS during agroinfiltration. When *A. tumefaciens* cells harboring an empty T-DNA plasmid containing the cauliflower mosaic virus (CaMV) 35S promoter were infiltrated into the leaves of *Nicotiana benthamiana* line 16c with a GFP gene over-expressed under the control of the same promoter, no small interfering RNAs (siRNAs) were derived from the GFP sequence. However, siRNAs derived from the CaMV 35S promoter were detected, indicating that TGS against the GFP gene was induced. When the GFP gene was inserted into the T-DNA plasmid, PTGS against the GFP gene was induced whereas TGS against the CaMV 35S promoter was suppressed. We also showed the importance of terminator sequences in T-DNA for gene silencing. Therefore, depending on the combination of promoter, terminator and coding sequences on T-DNA and the host nuclear genome, either or both TGS and/or PTGS could be induced by agroinfiltration. Furthermore, we showed the possible involvement of three siRNA-producing Dicers (DCL2, DCL3 and DCL4) in the induction of TGS by the co-agroinfiltration method. Especially, DCL2 was probably the most important among them in the initial step of TGS induction. These results are valuable for controlling gene expression by agroinfiltration.

## Introduction

RNA silencing is a form of sequence-specific gene silencing induced by small RNAs (sRNAs) of approximately 21–24 nucleotides (nt) and is widely conserved in eukaryotes (Baulcombe 2004). In plants, there are two major types of sRNAs, small interfering RNAs (siRNAs) and microRNAs (miRNAs), whose biogenesis is different. siRNA and miRNA pathways utilize different sets of key components for RNA silencing, i.e., Dicer-like (DCL) and Argonaute (AGO) proteins (Borges and Martienssen 2015; Fukudome and Fukuhara 2017). Different sized sRNAs generated by different DCLs are engaged in either transcriptional gene silencing (TGS) or post-transcriptional gene silencing (PTGS). The 24-nt siRNA produced by DCL3 is involved in TGS, which induces RNA-directed DNA methylation (RdDM) at the transcriptional promoter region and suppresses transcription itself (Matzke et al. 2015). During PTGS, 21-nt miRNAs generated by DCL1 and 21-nt and 22-nt siRNAs generated by DCL4 and DCL2, respectively, are involved in PTGS and guide AGOs to cleave target mRNAs that have complementary sequences of miRNAs and siRNAs. In other words, both TGS and PTGS function to silence target gene expression, although the molecular mechanisms in silencing target genes are distinctively different from each other. Since sRNAs play pivotal roles in both gene silencing mechanisms, both are collectively referred to as RNA silencing (Borges and Martienssen 2015).

RNA silencing is often induced when a foreign gene is introduced into a plant genome (Baulcombe 2004). *Agrobacterium tumefaciens* (*Rhizobium radiobacter*), a phytopathogenic bacterium, is used to introduce foreign genes into the plant nuclear genome because it has the ability to transfer a DNA fragment, called T-DNA, which is located in a tumor-inducing (Ti) plasmid, into a nuclear DNA of the infected plant (Gelvin 2003; Tzfira and Citovsky 2006). The *Agrobacterium*-mediated transient expression assay (agroinfiltration) is a convenient and popularly used method that allows for the over-expression of foreign genes in plant cells without creating stable transgenic plants (Johansen and Carrington 2001; Llave et al. 2000). However, RNA silencing can frequently be induced by this agroinfiltration method. For example, when *A. tumefaciens* cells harboring T-DNA containing the green fluorescent protein (GFP) gene driven by the cauliflower mosaic virus (CaMV) 35S promoter were infiltrated into the leaf of a transgenic *Nicotiana benthamiana* plant that over-expressed a GFP gene, GFP transcripts were sliced by the RNA-induced silencing complex (RISC), loading 21-nt siRNAs produced from a dsRNA form of GFP-transcripts by DCL4 (Borges and Martienssen 2015; Fukudome and Fukuhara 2017).

This RNA silencing (gene silencing) process can be easily monitored by the intensity of green fluorescence, so the agroinfiltration system consisted of GFP-over-expressing *A. tumefaciens* and GFP-over-expressing *N. benthamiana* line 16c is quite a useful and convenient method for inducing RNA silencing and evaluating RNA silencing factors such as viral suppressors of RNA silencing (VSRs) encoded by plant viruses (Burgyán and Havelda 2011; Roth et al. 2004). Indeed, numerous VSRs encoded by various viruses have been identified by this break-through agroinfiltration method since it was discovered (Llave et al. 2000; Johansen and Carrington 2001). Suppressor activities of VSRs against PTGS have mainly been reported so far, as evaluated by agroinfiltration, because PTGS, but not TGS, is thought to be a major defense mechanism of host plants against virus infections (Llave et al. 2000; Mallory et al. 2001; Marathe et al. 2000). Therefore, reports of the TGS induction by agroinfiltration are still limited (Philips et al. 2019). Since *N. benthamiana* is susceptible to many plant pathogens, including viruses and *A. tumefaciens* (Goodin et al. 2008), almost all agroinfiltration experiments have been performed by using *N. benthamiana* leaves. However, it is not suitable for genetic experiments (Goodin et al. 2008), and investigations on RNA silencing in *N. benthamiana* are largely delayed relative to those employing *A. thaliana*.

In this study, we attempted to induce PTGS of the GFP gene in GFP-expressing *N. benthamiana* line 16c by infiltrating leaves with *A. tumefaciens* cells harboring the GFP gene. We observed that GFP gene silencing was induced even when bacterial cells not harboring the GFP gene (i.e., harboring an empty vector as a negative control) were used. The expectation is that PTGS is induced when bacterial cells have T-DNA containing an over-expressing gene homologous to the expressed host gene, so it would be interesting if gene silencing were triggered by other conditions. We showed that the induction of TGS resulted in GFP gene silencing even when *A. tumefaciens* cells harboring a T-DNA plasmid as an empty vector were infiltrated. Furthermore, various T-DNA plasmids were used to characterize the structural features of T-DNA sequences under which TGS was induced during agroinfiltration. The results of this study will assist researchers to transiently express foreign genes introduced by agroinfiltration without interference by TGS, and to accurately control the induction of TGS and PTGS by agroinfiltration.

## Materials and methods

### Plant materials and growth conditions

Wild type (WT) and transgenic *N. benthamiana* plants were grown in pots in a room with a controlled environment under the following conditions: 50–100 µmol m^−2^ s^−1^, 16 h light and 8 h darkness, 24°C. Three transgenic *N. benthamiana* plants with knocked-down (KD) Dicer-like protein genes (*dcl2*-KD, *dcl3*-KD and *dcl4*-KD), respectively, by RNA interference (RNAi) were reported previously (Andika et al. 2015). Seeds of the *N. benthamiana* 16c line were kindly provided by Dr. David Baulcombe, the Sainsbury Laboratory, Norwich, UK (Ruiz et al. 1998).

### Preparation of plasmids for agroinfiltration

Two kinds of binary plasmids, pRI201-AN and pAT006, as well as their derivatives were used for agroinfiltration experiments (Supplementary Figure S1). pRI201-AN, which is commercially available from Takara Bio (Kusatsu, Japan), contains the CaMV 35S promoter and the heat shock protein (HSP) terminator derived from *Arabidopsis thaliana.* pAT006 has a pMDC background and contains the CaMV 35S promoter and terminator in the multiple cloning site (Tsuzuki et al. 2014). By cleaving pRI201-AN with two types of restriction endonucleases and self-ligating using DNA Ligation Kit Ver.2.1 (Takara Bio), four types of binary plasmids containing a shortened T-DNA region were prepared. The names of the prepared plasmids and the restriction enzymes used (in parentheses) are as follows: pRI-SA (*Sal*I and *Apa*I), pRI-XA (*Xba*I and *Apa*I), pRI-AH (*Apa*I and *Hind*III), and pRI-EH (*Eco*RI and *Hind*III). Structures of these plasmids are shown in Supplementary Figure S1. The DNA fragments of two kinds of GFP genes, enhanced GFP (eGFP) and soluble modified GFP (smGFP), which are derived from the plasmids, pEGFP-1 (Takara Bio) and pBICGFP (Davis and Vierstra 1998; Takeda et al. 2002), respectively, were cloned into pRI201-AN and pAT006 using the In-Fusion® HD Cloning kit or DNA Ligation Kit Ver.2.1 (Supplementary Figure S1).

### *Agrobacterium*-mediated transient expression assay (agroinfiltration)

**Figure 1:**
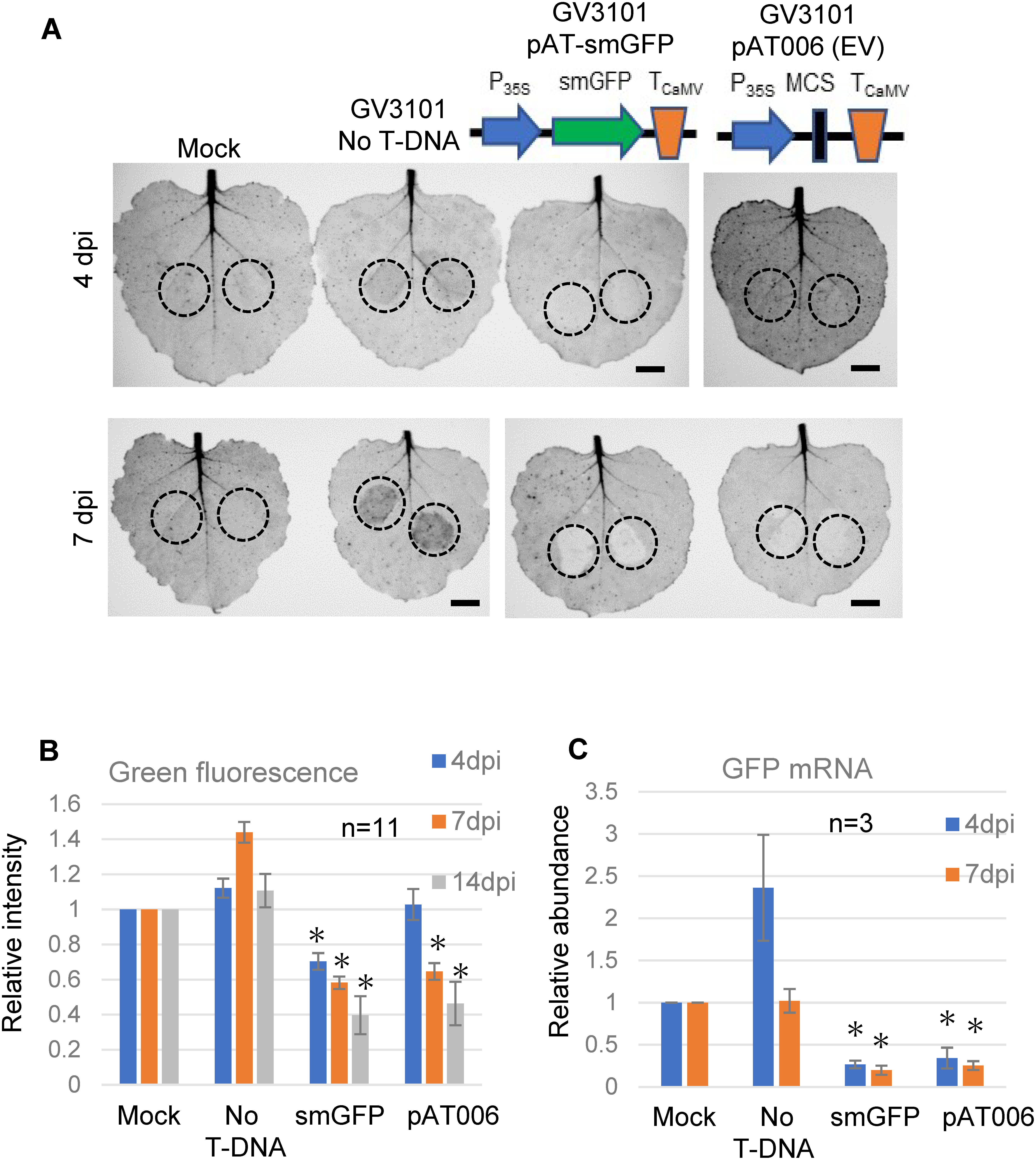
Figure 1. Induction of GFP gene silencing by infiltrating *A. tumefaciens* cells harboring the empty T-DNA plasmid as well as the GFP-expressing plasmid. (A) Photographs of *N. benthamiana* leaves over-expressing the GFP gene (16c line), into which *A. tumefaciens* GV3101 cells were infiltrated. Photographs were obtained by the imaging analyzer LAS-3000. Green fluorescence was observed at 4 and 7 dpi. Schematic drawings of T-DNA regions of pAT-smGFP and pAT006 (empty vector, EV) are shown. Mock indicates mock inoculation with agroinfiltration buffer, and broken circles indicate infiltrated sites. Bars indicate 1 cm. (B) Intensities of green fluorescence in infiltrated sites were analyzed with the image analysis software ImageJ (https://imagej.nih.gov/ij/) and their relative values are shown when the average intensity of mock-infiltrated sites was set to 1 as the standard. Bars indicate ±standard errors (SE) of 11 biological replicates. Significant differences were analyzed by Tukey’s test, and * indicates a significant difference (*p*<0.05). (C) Relative abundance of GFP transcript in each infiltrated spot at 4 and 7 dpi was measured by qPCR. Data were obtained from three biological replicates. The actin gene was used as the internal standard. The abundance of Mock was set to 1 as the standard. Bars indicate ±SE, and significant differences (Tukey’s test) are indicated by * (*p*<0.05).

Binary plasmids were introduced into *A. tumefaciens* (*R. radiobacter*) strain GV3101 (MP90) by the electroporation method using MicroPulser™ (Bio-Rad Laboratories, Hercules, CA, USA). Transformants were selected on the Luria Broth (LB) agar medium containing 20 µg ml^−1^ rifampicin, 20 µg ml^−1^ kanamycin and 30 µg ml^−1^ gentamycin at 28°C for 2 or 3 days. A transformed *Agrobacterium* colony was inoculated in LB liquid medium containing 20 µg ml^−1^ kanamycin and 30 µg ml^−1^ gentamycin at 28°C for 24 to 48 h. The culture solution was diluted with 50 volumes of the same LB medium and incubated for 16 to 24 h. Bacterial cells were harvested by centrifugation at 3,000 rpm for 15 min and suspended in agroinfiltration buffer (10 mM Mes-NaOH [pH 5.6] and 10 mM MgCl_2_). Bacterial cells were harvested by centrifugation, re-suspended in the agroinfiltration buffer containing 150 µM acetosyringone until OD_600_ was 0.5, incubated in the dark at 25°C for 3 to 5 h, then infiltrated into the leaves of WT, GFP-over-expressing (16c line) or dcl-KD *N. benthamiana* plants that were 4 to 6 weeks old. The intensities of green fluorescence in *N. benthamiana* leaves shown in color photographs (Figures 3–5 and Supplementary Figures S2–S4, S6 and S7) and black and white photographs (Figure 1 and Supplementary Figure S2) were measured by the Fluorescence Imaging System, FOBI (NeoScience, Seoul, South Korea) and the image analyzer LAS-3000 (FUJIFILM, Japan), respectively.

### Quantitative real-time PCR

Total RNA was isolated from agroinfiltrated spots of *N. benthamiana* leaves using the Trizol reagent following the manufacturer’s protocol (Thermo Fisher Scientific, Waltham, MA, USA), from which cDNAs were produced by the PrimeScript RT reagent Kit with gDNA Eraser (Takara Bio). Quantitative real-time PCR (qPCR) was performed by the Thermal Cycler Dice Real Time System (Takara Bio) with the GoTaq® qPCR Master Mix (Promega). Primers for qPCR were designed using the Primer3Plus program (http://www.bioinformatics.nl/cgi-bin/primer3plus/primer3plus.cgi/). Primer’s nucleotide sequences are shown in Supplementary Table S1.

### Detection of small RNAs (sRNAs)

Approximately 5 µg of total RNA isolated using the Trizol reagent was electrophoresed in 18% denaturing polyacrylamide gels containing 1×TBE buffer [89 mM Tris (pH 8.3), 89 mM boric acid, 2 mM EDTA] and 7 M urea, and blotted onto a nylon membrane (Zeta-Probe, Bio-Rad, USA) by electroblotting (1.0 mA/1.0 cm^2^ membrane for 1 h at 4°C). DNA fragments of the GFP gene and the CaMV 35S promoter as probes were amplified by PCR, and then probes for siRNA detection were made using the BcaBEST Labeling Kit (Takara Bio) with [α-^32^P]dCTP. PCR primers are listed in Supplementary Table S1. Hybridization was carried out in Perfect Hyb Plus hybridization buffer (Sigma-Aldrich) containing ^32^P-labeled DNA probe at 45°C for 6 h. Membranes were washed four times in 2×SSC (1×SSC, 0.15 M NaCl, 15 mM sodium citrate) with 0.5% SDS at 45°C for 1 h, and then analyzed by a Typhoon FLA 7000 image analyzer (GE Healthcare) (Fukuhara et al. 2011).

## Results

### Induction of GFP gene silencing by infiltrating *A. tumefaciens* cells harboring an empty T-DNA plasmid as well as a GFP-expressing plasmid

Agroinfiltration experiments were performed using the *N. benthamiana* 16c transgenic line, in which the GFP gene is constitutively expressed, and the *A. tumefaciens* GV3101 strain harboring various T-DNA plasmids, as well as those harboring the empty vector pAT006 as negative controls. We expected that infection by *A. tumefaciens* cells harboring pAT-smGFP containing the GFP gene would induce PTGS. As expected, the intensity of green fluorescence decreased significantly at the site infiltrated by bacterial cells harboring pAT-smGFP at 4 and 7 days post inoculation (dpi) (Figure 1A, B and Supplementary Figure S2). Unexpectedly, green fluorescence also decreased significantly at the site infiltrated by *A. tumefaciens* cells harboring the empty vector pAT006 at 7 dpi, even though it was used as a negative control (Figure 1A, B and Supplementary Figure S2). To examine whether the decrease in green fluorescence was caused by the reduction of GFP gene expression, the mRNA level of the GFP gene was quantified by qPCR (Figure 1C), which indicated that the level of GFP mRNA was reduced to approximately 20 to 30% following the infection of both bacterial cells harboring pAT-smGFP and the empty vector. Stated differently, the results showed that the reduction of green fluorescence in the infiltrated sites of bacterial cells containing the empty vector was caused by silencing of GFP gene expression.

A slight increase in green fluorescence and upregulation of GFP mRNA were also unexpectedly observed at the sites infiltrated by *A. tumefaciens* cells harboring no T-DNA plasmids (Figure 1 and Supplementary Figure S2). The cause of this upregulation of GFP mRNA is unknown, but the similar curious observation has been reported previously (Canto et al. 2002). A reflection on this phenomenon is provided in the Discussion.

### Induction of TGS by the T-DNA insertion

**Figure 2:**
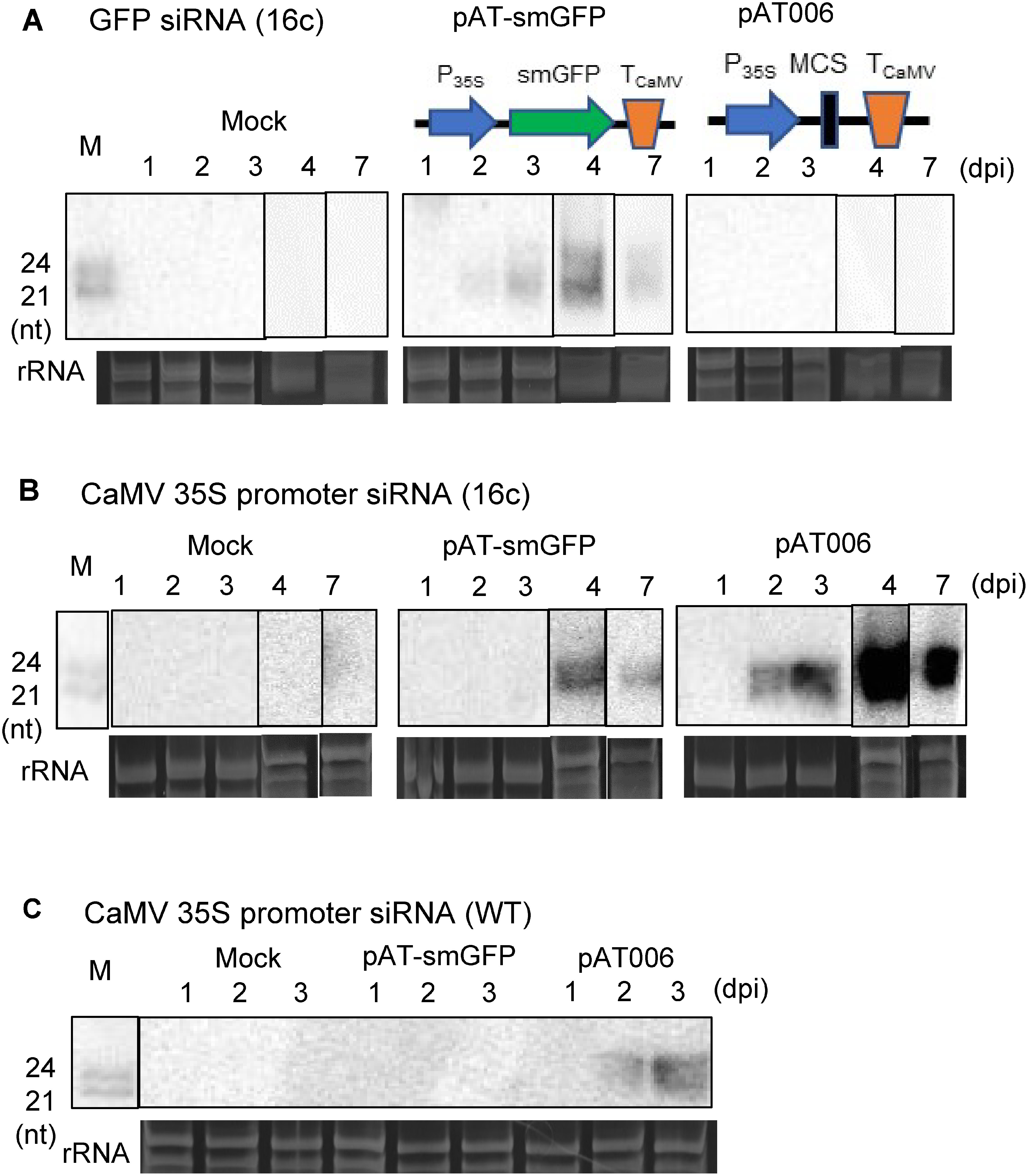
Figure 2. Induction of TGS by T-DNA insertion. Detection of siRNAs derived from the GFP gene (A) and the CaMV 35S promoter (B and C) by Northern hybridization using the full-length GFP fragment (A) or the CaMV 35S promoter fragment (B and C) as probes. After 1, 2, 3, 4 and 7 dpi, total RNA was isolated from the infiltrated sites of leaves of the *N. benthamiana* line 16c (A and B) or WT (C) plants. Schematic drawings of T-DNA regions of pAT-smGFP and pAT006 (empty vector, EV) are shown. M indicates molecular weight markers of 21-nt and 24-nt single-stranded RNAs (ssRNAs), and rRNA indicates rRNA bands stained by ethidium bromide as a loading control.

To investigate the cause of the decrease in green fluorescence and GFP mRNA in the infiltrated sites when *A. tumefaciens* cells harboring the empty vector pAT006 were inoculated into the leaves of GFP-expressing *N. benthamiana*, we attempted to detect siRNAs derived from the GFP coding sequence by Northern hybridization (Figure 2 and Supplementary Figure S3). The accumulation of GFP siRNAs was confirmed at the site infiltrated by bacterial cells harboring the pAT-smGFP plasmid, but not at the sites infiltrated by bacterial cells harboring the empty plasmid pAT006 (Figure 2A). Thus, even though GFP siRNAs were not detected, the amount of GFP mRNA decreased at the sites infiltrated by bacterial cells harboring pAT006. We hypothesize that the induction of TGS by the CaMV 35S promoter caused GFP gene expression to be silenced because the CaMV 35S promoter is commonly to both the nuclear DNA of the *N. benthamiana* 16c line and the T-DNA region of pAT006. Indeed, large amounts of siRNAs of 21 to 24 nt derived from the CaMV 35S promoter were detected from the infiltrated sites of the pAT006-harboring strain (Figure 2B). The similar results have been reported previously (Canto et al. 2002). A much smaller amount of CaMV 35S promoter-derived siRNA was also detected in plants agroinfiltrated by bacterial cells carrying the smGFP gene (pAT-smGFP). These results indicate that TGS was induced when the T-DNA sequence containing the 35S promoter was introduced into the host nucleus that carries the 35S promoter, and that PTGS was also induced when the T-DNA sequence contains a homologous sequence (i.e., the GFP sequence) to a transcribing sequence in the host nuclear DNA. Furthermore, even when a similar experiment was performed using a WT *N. benthamiana* plant, the accumulation of siRNAs derived from the CaMV 35S promoter sequence was detected at the sites infiltrated by bacterial cells harboring pAT006 (Figure 2C), indicating that siRNAs from the CaMV 35S promoter in T-DNA can be produced not only in the 16c line, which has the CaMV 35S promoter in nuclear DNA, but also in WT *N. benthamiana* plants. As compared between Figures 2B and 2C, siRNAs derived from the 35S promoter in WT accumulated less than that in 16c.

### Structural features of T-DNA to induce TGS

**Figure 3:**
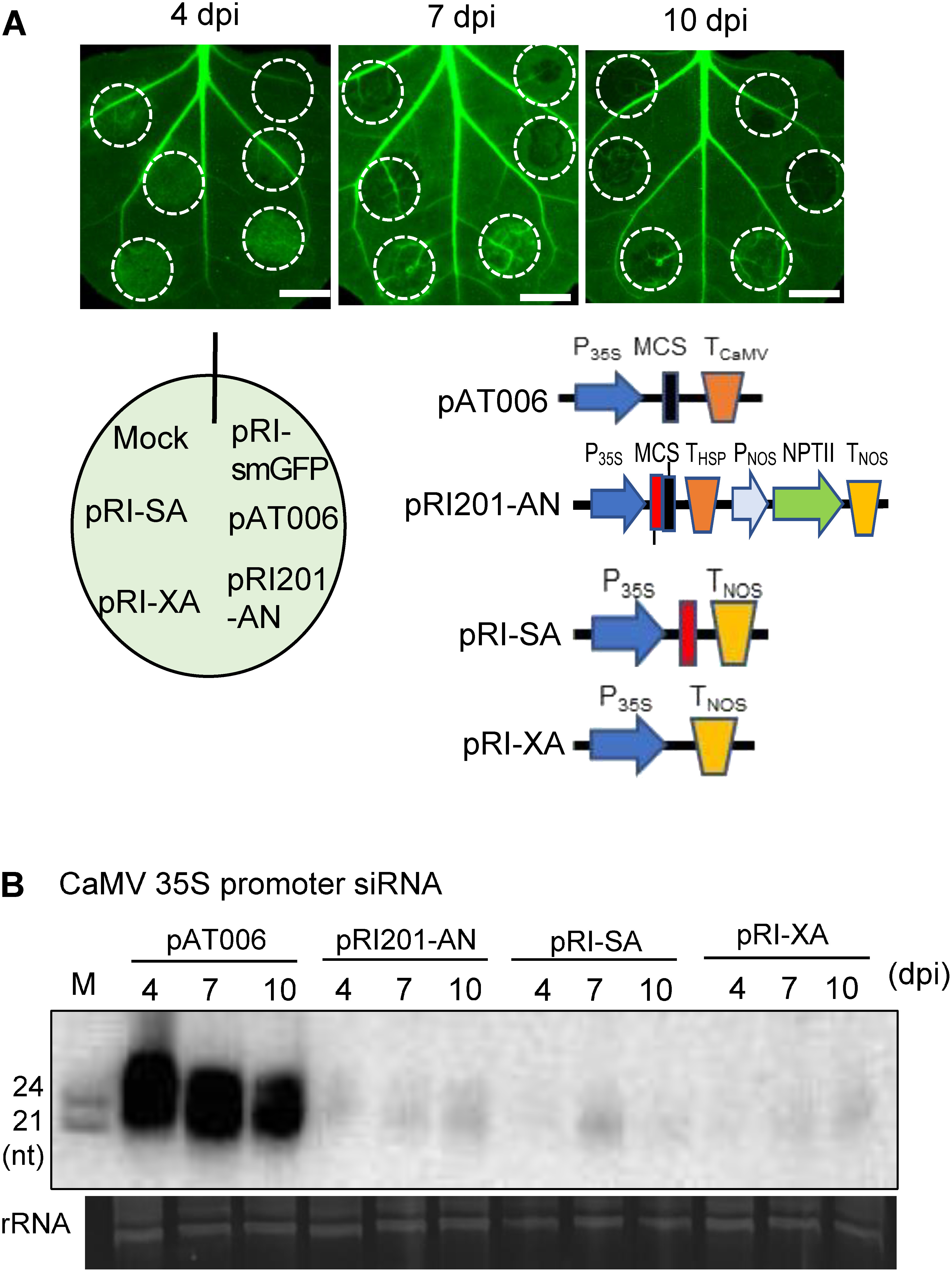
Figure 3. The structural feature of T-DNA to induce TGS. (A) Photographs of *N. benthamiana* leaves over-expressing the GFP gene (16c line), into which *A. tumefaciens* cells were infiltrated. Structures of plasmids are shown in Supplementary Figure S1. Green fluorescence was observed at 4, 7 and 10 dpi. White broken circles indicate infiltrated sites, and bars indicate 1 cm. (B) Detection of siRNAs derived from the CaMV 35S promoter by Northern hybridization. Total RNA was prepared from the infiltrated sites. Schematic drawings of T-DNA regions of four plasmids that were used in this experiment are shown. M indicates molecular weight markers of 21-nt and 24-nt ssRNAs, and rRNA indicates rRNA bands stained by ethidium bromide as a loading control.

Unlike pAT006, which readily induced TGS, as was shown above, another commercially available plasmid pRI201-AN, which has the CaMV 35S promoter, required more than 10 days to silence green fluorescence when *A. tumefaciens* cells harboring this plasmid were inoculated into the leaves of GFP-expressing *N. benthamiana* 16c plants (Figure 3A and Supplementary Figure S4). The difference between these two empty vectors, pAT006 and pRI201-AN, lies in the lengths of their T-DNA regions (1.0 and 3.6 kbp, respectively), which are comprised of promoter, protein-coding and terminator sequences (Figure 3B and Supplementary Figure S1). Therefore, in order to investigate how these components affect the induction of TGS, plasmids containing various T-DNA sequences were prepared by modifying pRI201-AN (Supplementary Figure S1). pRI-XA and pRI-SA with reduced T-DNA size (1.6 and 1.7 kbp, respectively) were prepared by deleting some sequences from the pRI201-AN plasmid (Figure 3B and Supplementary Figure S1). The reduction of GFP fluorescence was observed at 7 dpi with pRI-SA and at 10 dpi with pRI-XA, which was earlier than when pRI201-AN was used (Figure 3A and Table 1). siRNAs derived from the 35S promoter were detected from the sites infiltrated by bacterial cells harboring pRI201-AN, pRI-SA or pRI-XA, but the amount of siRNAs was much less than from cells carrying pAT006 (Figure 3B). These results show that the induction of TGS by inoculation with bacterial cells harboring an empty vector is a common phenomenon, and that the time required for TGS induction can be shortened by reducing the length of T-DNA.

**Table 1:** Table 1. Summary of the induction of TGS and PTGS by pAT006, pRI201-AN and their derivatives.

Plasmid name	Plasmid size (kbp)	T-DNA size (kbp)	T-DNA			GF disappear (dpi)	GFP siRNA	35S siRNA
			promoter	insert	terminator			
pAT006	6.5	1.0	35S	—	CaMV	5	—	+++
pAT-smGFP	7.2	1.7	35S	smGFP	CaMV	3	++	+
pRI201-AN	10.4	3.6	35S	—	HSP	12–14	—	+
pRI-smGFP	11.1	4.3	35S	smGFP	HSP	4–7	+	—
pRI-eGFP	11.1	4.3	35S	eGFP	HSP	14∼	+	—
pRI-SA	8.5	1.7	35S	—	NOS	7	ND	+
pRI-XA	8.4	1.6	35S	—	NOS	10	ND	+
pRI-EH	9.1	2.3	NOS	—	NOS	14	ND	—
pRI-AH	7.5	0.7	—	—	NOS	—	ND	—

GF, green fluorescence; ND, not determined; —, no or not detected; +, detected.

In order to investigate the role of the CaMV 35S promoter in T-DNA during TGS induction, two plasmids, pRI-EH and pRI-AH, which were prepared by removing the 35S promoter sequence from T-DNA, were used in agroinfiltration experiments. GFP fluorescence at the infiltration sites was maintained for a long time, and no siRNAs derived from the 35S promoter were detected (Supplementary Figure S4, Table 1). In other words, short T-DNAs without the CaMV promoter failed to induce TGS of the CaMV 35S promoter.

From the viewpoint of transcriptional terminator sequences, pAT006 containing the CaMV-derived terminator strongly induced TGS by the action of many siRNAs derived from the 35S promoter, starting from the earliest day of infiltration (Figure 1 and Supplementary Figure S1). This was followed by pRI-SA and pRI-XA, which contain the terminator derived from the *Agrobacterium* nopaline synthase (NOS) gene (Figure 3 and Supplementary Figure S1). Finally, induction of TGS by pRI201-AN, which has the terminator derived from the *Arabidopsis* HSP gene, took the most time (Figure 3 and Supplementary Figure S1). These results might support previous claims that terminators play a key role in the expression and PTGS of transgenes (de Felippes et al. 2020) and that the *Arabidopsis* HSP terminator increases the expression of transgenes in plant cells (de Felippes et al. 2022; Nagaya et al. 2010; Pérez-González and Caro 2018), i.e., the induction level of TGS may depends on the origin of terminators.

### Structural features of T-DNA to suppress TGS

**Figure 4:**
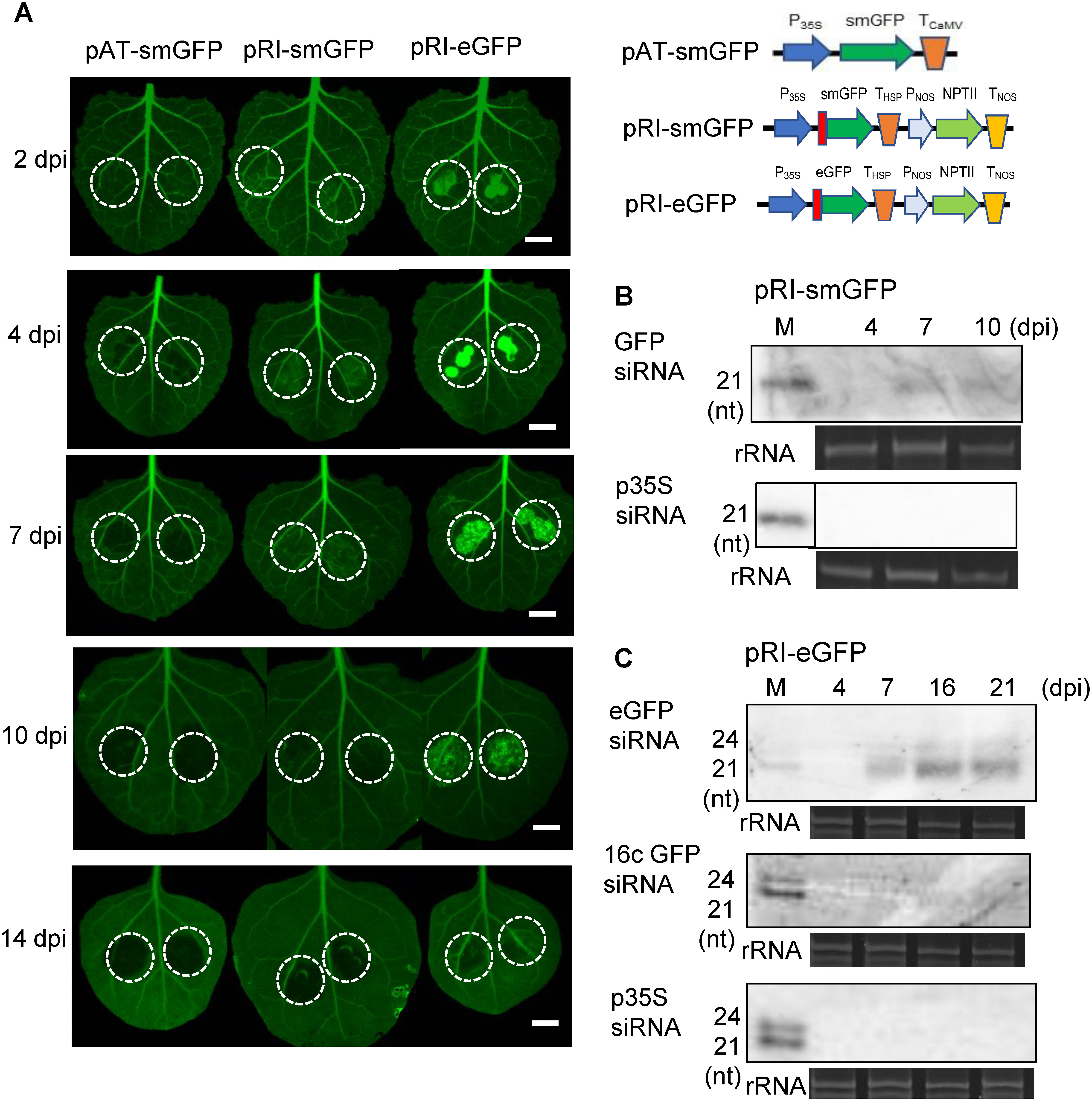
Figure 4. The structural feature of T-DNA to suppress TGS induction. (A) Photographs of *N. benthamiana* 16c leaves, into which *A. tumefaciens* cells were infiltrated. Green fluorescence was observed at 2, 4, 7, 10 or 14 dpi. Schematic drawings of T-DNA regions of three GFP-expressing plasmids are shown. White broken circles indicate infiltrated sites, and bars indicate 1 cm. (B) Detection of siRNAs derived from either the smGFP coding sequence in T-DNA or the 16c GFP coding sequence in nuclear DNA (GFP siRNA, PTGS) and the CaMV 35S promoter (p35S siRNA, TGS) by Northern hybridization. Since the nucleotide sequence of smGFP coding region in T-DNA is very similar (approximately 95%) to that of the nuclear GFP coding sequence in the *N. benthamiana* 16c plant, siRNAs derived from both GFP genes is detectable by the smGFP probe. Total RNA was isolated from the infiltrated sites of the *N. benthamiana* 16c leaves, into which *A. tumefaciens* cells containing pRI-smGFP were infiltrated, at 4, 7 and 10 dpi. (C) Detection of siRNAs derived from the eGFP coding sequence in T-DNA (PTGS), the 16c GFP coding sequence in nuclear DNA (PTGS) and the CaMV 35S promoter (TGS) by Northern hybridization. Since the nucleotide sequence of eGFP (Accession No. U55761) in T-DNA is approximately 75% different from that of the nuclear 16c GFP (Accession No. U70495), siRNAs derived from eGFP and 16c GFP could be distinguished by Northern hybridization. After 4, 7, 16 and 21 dpi, total RNA was isolated from the sites of the *N. benthamiana* line 16c leaves into which *A. tumefaciens* cells containing pRI-eGFP were infiltrated. M indicates molecular weight markers of 21-nt and 24-nt ssRNAs, and rRNA indicates rRNA bands stained by ethidium bromide as a loading control.

To examine the structural features of T-DNA that induce or suppress TGS and/or PTGS, two GFP sequences, smGFP and eGFP (which emits stronger fluorescence than smGFP), were inserted into pRI201-AN to construct pRI-smGFP and pRI-eGFP, respectively (Figure 4 and Supplementary Figure S1). Agroinfiltration experiments with *N. benthamiana* 16c plants were carried out using these two plasmids as well as pAT-smGFP as the control. The results using pAT-smGFP are shown in Figures 1 and 2.

At the infiltration sites of pRI-smGFP-harboring cells, small amounts of GFP siRNAs derived from T-DNA and/or nuclear DNA, but not siRNAs derived from the CaMV 35S promoter, were detected at 7 and 10 dpi (Figure 4B), indicating that PTGS of the GFP gene was induced whereas no TGS to the 35S promoter was induced. The empty plasmid pAT006 strongly induced TGS of the 35S promoter (Figure 1) and large amounts of siRNAs derived from the CaMV 35S promoter were detected (Figures 2B and 3B). Whereas pAT-smGFP (pAT006 containing the GFP sequence) still weakly induced TGS of the promoter because small amounts of 35S siRNAs were detected (Figure 2B). Similarly, the empty plasmid pRI201-AN weakly induced TGS of the 35S promoter (Figure 3) whereas pRI-smGFP (pRI201-AN containing the GFP coding sequence) did not induce TGS (Figure 4B). Therefore, TGS of the 35S promoter was induced by both empty plasmids with different origins (see Supplementary Figure S1), and the insertion of the GFP coding sequence into these empty plasmids suppressed the induction of TGS (Figures 2 and 4, Table 1).

The intensity of green fluorescence at the infiltration sites of the pRI-smGFP-harboring cells was slightly stronger and maintained longer than that at the infiltration sites by the pAT-smGFP-harboring cells (Figure 4A, Supplementary Figure S5 and Table 1). Since pRI-smGFP contains the HSP terminator whereas pAT-smGFP contains the CaMV-derived terminator, these results support the possibility that the induction of TGS and/or PTGS was suppressed by the *Arabidopsis* HSP terminator.

The induction of GFP gene silencing required more time at the infiltration sites of *A. tumefaciens* cells harboring pRI-eGFP than at the infiltration sites of cells harboring pRI-smGFP (Figure 4A, Supplementary Figure S5 and Table 1). Since there is 75% nucleotide sequence identity between eGFP and smGFP, which the 16c plant also contains in its nuclear DNA, Northern hybridization using the eGFP-specific probe was performed to detect the eGFP-specific siRNAs. The results show that mainly 21 nt of eGFP siRNA accumulated but neither siRNAs derived from the 35S promoter nor those from the nuclear GFP (16c GFP) coding sequence were detected (Figure 4C). These results suggest that PTGS for the T-DNA-derived eGFP coding sequence was mainly induced at the inoculated site by eGFP-harboring cells but that neither TGS of the 35S promoter nor PTGS of the nuclear GFP coding sequence was induced.

### Involvement of siRNA-producing Dicers for the induction of TGS

**Figure 5:**
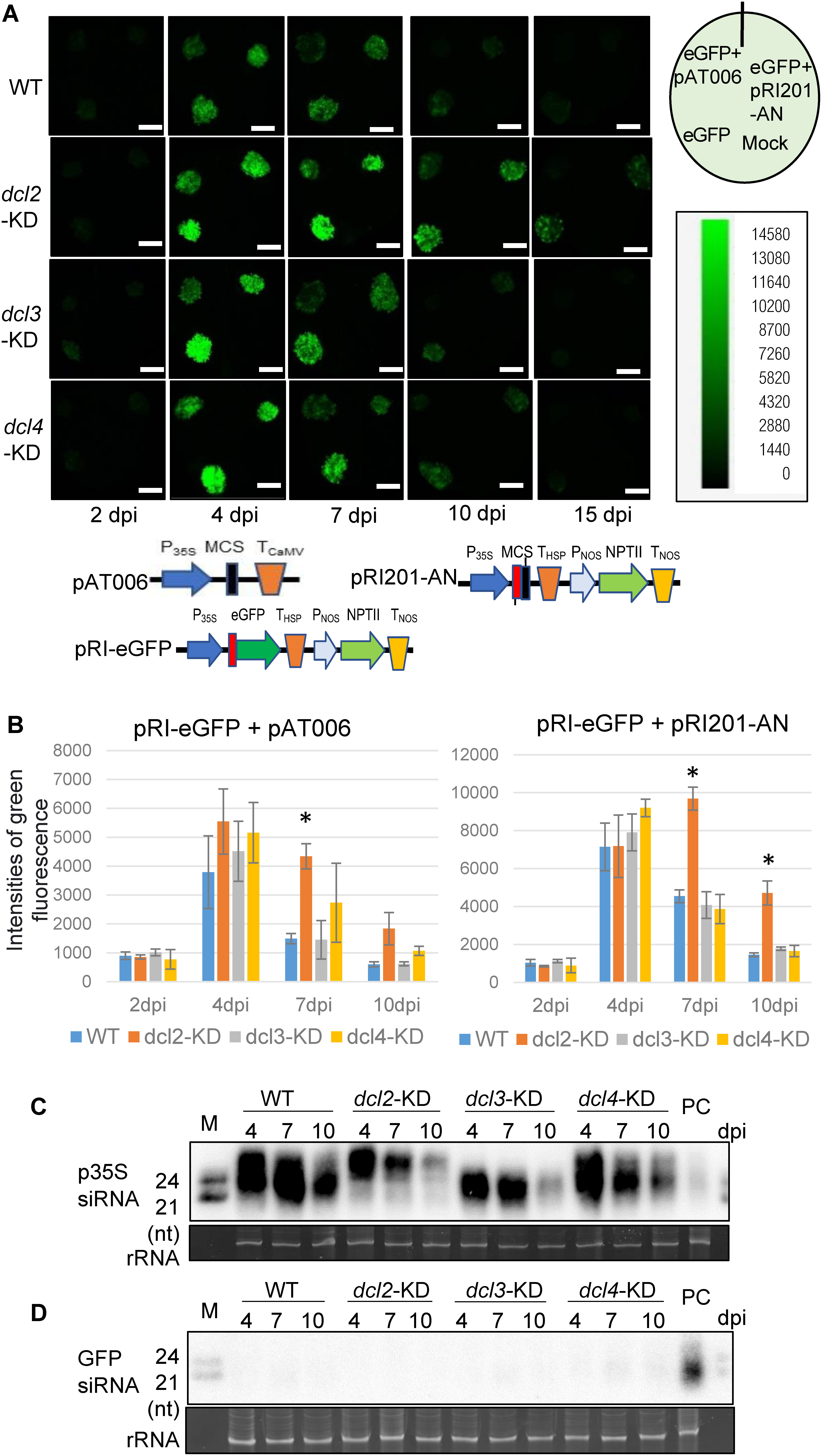
Figure 5. Involvement of siRNA-producing Dicers for TGS induction. (A) Photographs of leaves of WT and three *dcl*-KD mutant plants of *N. benthamiana*, into which *A. tumefaciens* cells harboring T-DNA plasmids were co-infiltrated. *A. tumefaciens* cells harboring pRI-eGFP was mixed with either *A. tumefaciens* cells harboring pAT006 or those harboring pRI201-AN, and then co-infiltrated into leaves of WT *N. benthamiana* plants. Only *A. tumefaciens* cells harboring pRI-eGFP was also infiltrated as a control, and Mock indicates mock inoculation with agroinfiltration buffer. Schematic drawings of T-DNA regions of three plasmids are shown. Green fluorescence was observed at 2, 4, 7, 10 and 15 dpi by the FOBI system. Bars indicate 1 cm. (B) Intensities of green fluorescence in infiltrated sites were quantified and analyzed by the NEO image program (pRI-eGFP+pAT006 and pRI-eGFP+pRI201-AN). Bars indicate ±standard errors (SE) of four biological replicates, and significant differences (Tukey’s test) are indicated by * (*p*<0.01). (C) and (D) Detection of siRNAs derived from the 35S promoter (C) and derived from the GFP coding sequence (D) from co-infiltrated sites of *A. tumefaciens* cells harboring pRI-eGFP and those harboring pAT006 by Northern hybridization. After 4, 7 and 10 dpi, total RNA was isolated from the infiltrated sites of leaves of WT and three *dcl*-KD mutant plants of *N. benthamiana*, into which *A. tumefaciens* cells harboring pRI-eGFP and pAT006 were co-infiltrated. M indicates molecular weight markers of 21-nt and 24-nt ssRNAs, and rRNA indicates rRNA bands stained by ethidium bromide as a loading control. PC in (C) and (D) indicates as a positive control of siRNAs that were detected at the infiltrated site of the *N. benthamiana* 16c leaf by *A. tumefaciens* cells harboring pAT-smGFP, which are shown in Figure 2A, B.

We investigated the involvement of three siRNA-producing Dicers (DCL2, DCL3 and DCL4) in the induction of TGS by co-agroinfiltration using three transgenic *N. benthamiana* plants, in each of which one of the three DCL genes was respectively knocked-down (KD) by RNAi (Supplementary Figure S6) (Andika et al. 2015). The *A. tumefaciens* cells harboring the T-DNA plasmid containing the eGFP gene driven by the CaMV 35S promoter (pRI-eGFP) and those harboring the empty T-DNA plasmid (pAT006 or pRI201-AN) were mixed in equal amounts and co-infiltrated into the leaves of three *dcl*-KD and WT *N. benthamiana* plants, and the bacterial cells harboring pRI-eGFP were also used to singly infiltrate as a control (Figure 5A and Supplementary Figures S7 and S8). Strongest green fluorescence was observed at 4 dpi, similar to the results shown in Figure 4A (pRI-eGFP), and a decrease of green fluorescence at 7 to 15 dpi indicated the induction of GFP gene silencing (Figure 5A, B and Supplementary Figures S7–S9). Large amounts of siRNAs derived from the 35S promoter were detected in the co-infiltrated sites by bacterial cells harboring pRI-eGFP and pAT006 in leaves of all four plants examined (Figure 5C) but few siRNAs derived from the GFP coding sequence were detected (Figure 5D), indicating that TGS against the CaMV 35S promoter, but not PTGS against GFP mRNA, was a main cause for GFP gene silencing at co-infiltration sites. The accumulation patterns of 21- to 24-nt siRNAs derived from the 35S promoter sequence were different among the four plant lines and specific-sized siRNAs decreased appropriately in the specific *dcl*-KD plants (Figure 5C), indicating that all three DCLs produced siRNAs derived from the 35S promoter sequence. This conclusion is supported by the Northern hybridization results shown in Figures 2B and 3B. These results indicate the possible involvement of three Dicers in the induction of TGS. Furthermore, higher green fluorescence was maintained at 7 dpi in *dcl2*-KD than in WT plants (Figure 5A, B and Supplementary Figure S8), indicating that DCL2 was possibly the most important among them in the induction of TGS. Since it has been reported that the non-canonical RdDM pathway containing DCL2 and DCL4 is involved in establishing initial DNA methylation at new target loci (Marí-Ordóñez et al. 2013; Matzke et al. 2015; Nuthikattu et al. 2013). In this co-agroinfiltration system, DCL2, rather than DCL3, is probably involved in the initial step of TGS induction.

## Discussion

**Figure 6:**
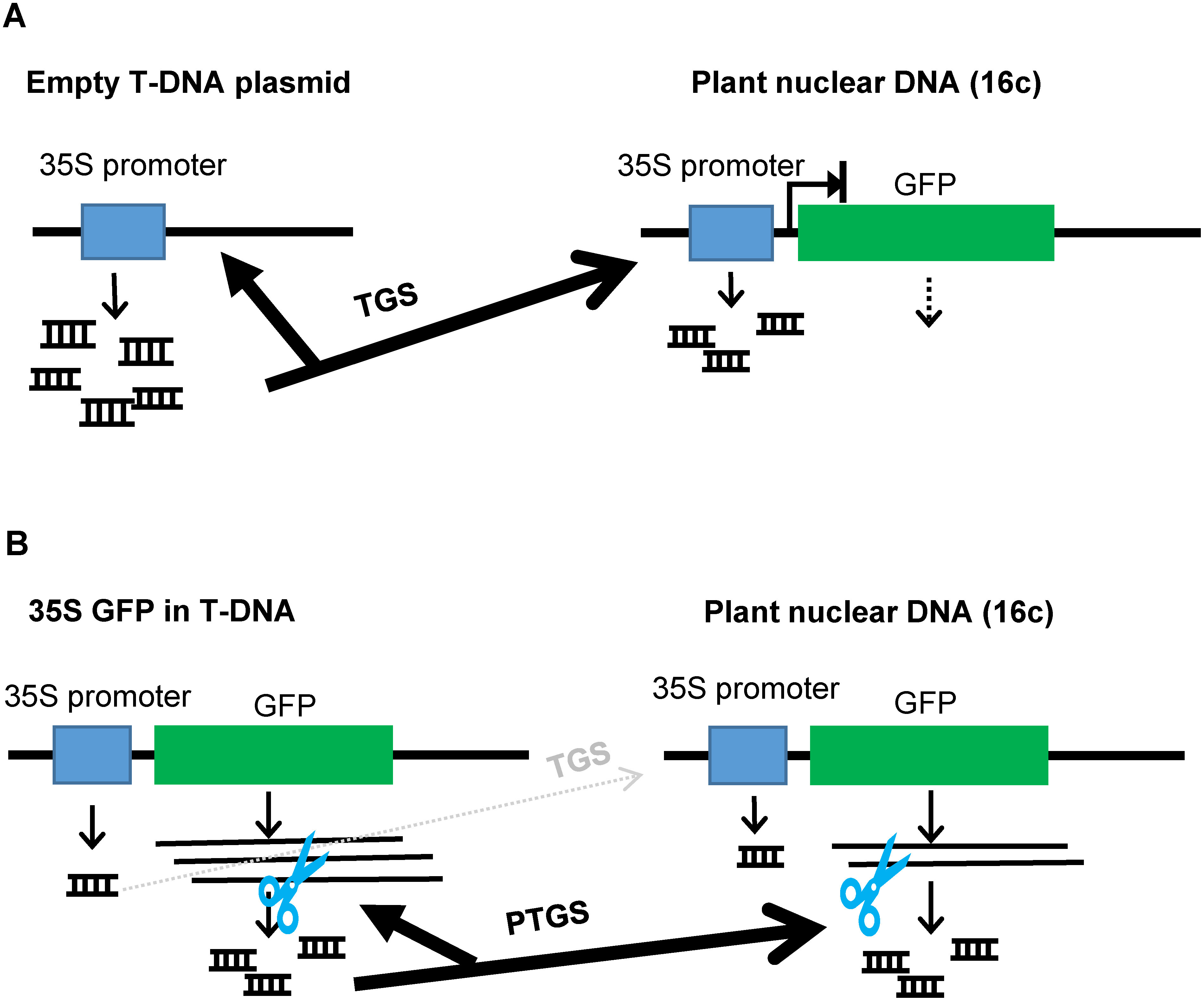
Figure 6. Graphical abstract. Schematic drawing of the induction of TGS and PTGS by agroinfiltration. (A) When *A. tumefaciens* cells harboring an empty T-DNA plasmid containing the CaMV 35S promoter were infiltrated into leaves of a transgenic *N. benthamiana* plant that has the GFP gene with the CaMV 35S promoter in its nuclear DNA (e.g., 16c line), GFP gene silencing was induced but no siRNAs derived from the GFP coding sequence (PTGS) are detected while those derived from the 35S promoter (TGS) were detected. (B) When the GFP coding sequence was inserted into the T-DNA plasmid, PTGS against the GFP coding sequence was mainly induced but TGS against the CaMV 35S promoter was suppressed.

PTGS against the GFP gene is thought to be induced by agroinfiltration using GFP-overexpressing plants (e.g., the *N. benthamiana* 16c line) and bacterial cells harboring GFP-overexpressing T-DNA. For this reason, many studies have used agroinfiltration to evaluate the activity of VSRs as the inhibitor activity for PTGS against the GFP gene (Burgyán and Havelda 2011; Llave et al. 2000; Roth et al. 2004). Here we showed that TGS against the CaMV 35S promoter as well as PTGS against the GFP coding sequence were induced depending on the structure of T-DNA (Figures 1–5). We also characterized the structural features of T-DNA that induce TGS. Namely, in the case of an empty vector in which only the CaMV 35S promoter and the terminator derived from CaMV were present in the T-DNA, TGS against the 35S promoter was strongly induced (Figure 6). Conversely, the inclusion of an expressing gene with the plant-derived HSP terminator in T-DNA suppressed TGS induction, which is consistent with previous reports (de Felippes et al. 2022, 2020; Nagaya et al. 2010). Our results provide useful information for the stable transient expression of any foreign genes and the assay of suppressor activity of VSRs by using agroinfiltration. In particular, the results showing that TGS was easily induced by agroinfiltration using bacterial cells harboring an empty vector, which is often used as a negative control, is useful for evaluating experimental results obtained by agroinfiltration (Figure 6 and Table 1).

Fairly recently, it was reported that DNA methylation is induced at the CaMV 35S promoter in T-DNA introduced into plant nuclear DNA by agroinfiltration at 2 to 3 dpi, reaching a maximum at about 7 dpi (Philips et al. 2019). In this study, the accumulation of siRNA derived from the CaMV 35S promoter reached a maximum at 4–7 dpi, indicating that the accumulation of siRNA must be correlated with the induction of DNA methylation and suppression of transcription (Figures 2 and 3). That is, siRNAs are generated from the promoter sequence (e.g., CaMV 35S promoter) in T-DNA when T-DNA is inserted into the plant nuclear DNA by agroinfiltration (Figures 2 and 3), de novo DNA methylation is induced in the promoter sequence in T-DNA and its homologous sequence(s) in nuclear DNA (Philips et al. 2019), and finally TGS against the following genes is induced.

Virus (CaMV)- or bacterium (*Agrobacterium*)-derived promoters and terminators are often used to control the expression of exogenous genes in agroinfiltration and transgenic plants. However, these virus- and prokaryote-derived sequences are likely to induce siRNA production, causing RNA silencing (Canto et al. 2002; de Felippes et al. 2020). pRI201-AN is reportedly used for the high expression of transgene products in plants by using the *Arabidopsis* HSP gene-derived terminator (de Felippes et al. 2022; Nagaya et al. 2010; Pérez-González and Caro 2018). This study also showed that the selection of a terminator is important for the suppression of TGS induction, leading to stable expression of a foreign gene.

The T-DNA portion of pAT006, which strongly induced TGS, is approximately 1.0 kbp in size (Supplementary Figure S1). We also found that shortening the size of the T-DNA segment of pRI201-AN shortened the time required for TGS induction (Figure 3A, B and Supplementary Figure S1). It is speculated that multiple copies of T-DNA are inserted into the host nuclear DNA to induce TGS when the T-DNA size is short. This speculation is supported by the results shown in Figure 2C, which shows that 35S promoter-derived siRNAs were detected in WT *N. benthamiana* leaves by infiltrating bacterial cells harboring pAT006. However, as far as we know, no reports investigating the correlation between T-DNA size and copy number inserted into the host nuclear DNA have been published. In both pAT006 and pRI201-AN, TGS was induced against the 35S promoter in the empty plasmid, but the induction of TGS was suppressed by inserting the GFP gene. This result suggests that transcripts of untranslatable RNAs from empty vectors induce TGS.

The canonical RdDM pathway (the most well-characterized RdDM pathway to date), which is composed of RNA polymerase IV (Pol IV), RNA-dependent RNA polymerase 2 (RDR2) and DCL3, acts to maintain and reinforce existing DNA methylation patterns (Matzke et al. 2015). Study on active transposons has revealed variations of the RdDM pathway, referred to as non-canonical RdDM (Marí-Ordóñez et al. 2013; Nuthikattu et al. 2013). Unlike canonical RdDM, the non-canonical RdDM pathway containing DCL2 and DCL4 is involved in establishing initial DNA methylation at new target loci, like novel transposon insertions (Marí-Ordóñez et al. 2013; Nuthikattu et al. 2013). Co-agroinfiltration experiments showed that the empty vector pAT006 also induced TGS during co-agroinfiltration (Figure 5). In addition, in this experimental system, DCL2 and DCL4, as well as DCL3, were shown to be involved in the production of CaMV 35S promoter-derived siRNAs (Figure 5C). It is interesting and noteworthy that strong green fluorescence was maintained at 7 dpi in *dcl2*-KD (Figure 5A, B). Therefore, in this co-agroinfiltration system, the non-canonical RdDM, in which DCL2 plays an important role, is probably involved in the initial step of TGS induction.

Agrobacterium infection has been reported to inhibit the RNA silencing pathway of host plant cells (Dunoyer et al. 2006; Pumplin and Voinnet 2013). In this study, green fluorescence and the accumulation of GFP mRNA increased when inoculated with *A. tumefaciens* cells without a T-DNA plasmid (Figure 1 and Supplementary Figure S2). Canto et al. (2002) has been reported the similar phenomenon two decades ago. These results suggest that *A. tumefaciens* infection inhibited host RNA silencing against GFP gene over-expression in *N. benthamiana* 16c plants, leading to an increase in green fluorescence and an increase in GFP mRNA accumulation (Figure 1 and Supplementary Figure S2).

## Abbreviations

AGO: Argonaute
CaMV: cauliflower mosaic virus
DCL: Dicer-like
dpi: days post inoculation
eGFP: enhanced GFP
EV: empty vector
GFP: green fluorescent protein
HSP: heat shock protein
KD: knocked-down
LB: Luria Broth
miRNA: microRNAs
NOS: nopaline synthase
nt: nucleotides
Pol IV: RNA polymerase IV
PTGS: post-transcriptional gene silencing
qPCR: quantitative real-time PCR
RdDM: RNA-directed DNA methylation
RDR2: RNA-dependent RNA polymerase 2
RISC: RNA-induced silencing complex
RNAi: RNA interference
siRNA: small interfering RNAs
smGFP: soluble modified GFP
sRNA: small RNAs
ssRNA: single-stranded RNAs
TGS: transcriptional gene silencing
Ti: tumor-inducing
VSR: viral suppressor of RNA silencing
WT: wild type

## Description of Supplementary Files

Supplementary Table S1. Primer list for PCR analysis.

Supplementary Figure S1. Schematic drawings of T-DNA plasmids used in agroinfiltration.

Supplementary Figure S2. Related to Figure 1. Photographs of *N. benthamiana* leaves over-expressing the GFP gene, into which *A. tumefaciens* cells were infiltrated.

Supplementary Figure S3. Related to Figure 2. Photographs of *N. benthamiana* leaves of WT and 16c line, into which *A. tumefaciens* cells were infiltrated.

Supplementary Figure S4. Related to Figure 3. Photographs of *N. benthamiana* leaves of 16c line and autoradiography of Northern hybridization.

Supplementary Figure S5. Related to Figures 3 and 4. Photographs of *N. benthamiana* leaves over-expressing the GFP gene (16c line), into which *A. tumefaciens* cells were infiltrated.

Supplementary Figure S6. Related to Figure 5. Confirmation of knocked-down of NbDCL genes in RNAi *N. benthamiana* plants by qPCR.

Supplementary Figure S7. Related to Figure 5. The induction of TGS to the GFP gene in a WT *N. benthamiana* plant by co-agroinfiltration.

Supplementary Figure S8. Related to Figure 5. Photographs of leaves of WT and three *dcl*-KD mutant plants of *N. benthamiana*, into which *A. tumefaciens* cells harboring T-DNA plasmids were co-infiltrated.

Supplementary Figure S9. Related to Figure 5. Intensities of green fluorescence in infiltrated sites were quantified and analyzed by the NEO image program.
