# Supplementary material for: Structural features of T-DNA that induce transcriptional gene silencing during agroinfiltration
Source: Plant Biotechnol (Tokyo). 2023 Dec 25;40(4):289–99. doi: 10.5511/plantbiotechnology.23.0719a (PMC10905568; doi:10.5511/plantbiotechnology.23.0719a)
Supplement: Supplementary Data [file plantbiotechnology-40-4-23.0719a-s001.pdf]

Structural features of T-DNA that induce transcriptional gene silencing during agroinfiltration

Supplementary Files

Supplementary Table S1 Nucleotide sequences of PCR primers.

Primer name	Nucleotide sequence (5′-3′)
For qPCR	
NbACT_F	AAAGACCAGCTCATCCGTGG
NbACT_R	CATGAATGCCAGCAGCTTCC
16cGFP_F	GCGATGGCCCTGTCCTTTTA
16cGFP_R	TGCCATGTGTAATCCCAGCA
NbDCL2_F	TTGCTTCCATCTTTGGGTTC
NbDCL2_R	CGTTTGTGACCTTGCGTAGA
NbDCL3_F	ATCTCCCATCGTGACTCCTG
NbDCL3_R	TCAGCAACCAGCAACTCATC
NbDCL4_F	AGGAAACCCCAGAAGAGCAT
NbDCL4_R	CCGCAGTATGTCCCAACTTT
For Probes	
35S promoter_F	ACAAAGGGTAATATCCGGAAACCT
35S promoter_R	AGAGGAAGGGTCTTGCGAAG
16cGFPprobe_F	ATGAGTAAAGGAGAAGAACTTTTCA
16cGFPprobe_R	TTATTTGTATAGTTCATCCATGCCA
eGFP_F	AGGGTACCATGGTGAGCAAG
eGFP_R	TTTGAACGATCGGGGAAAT

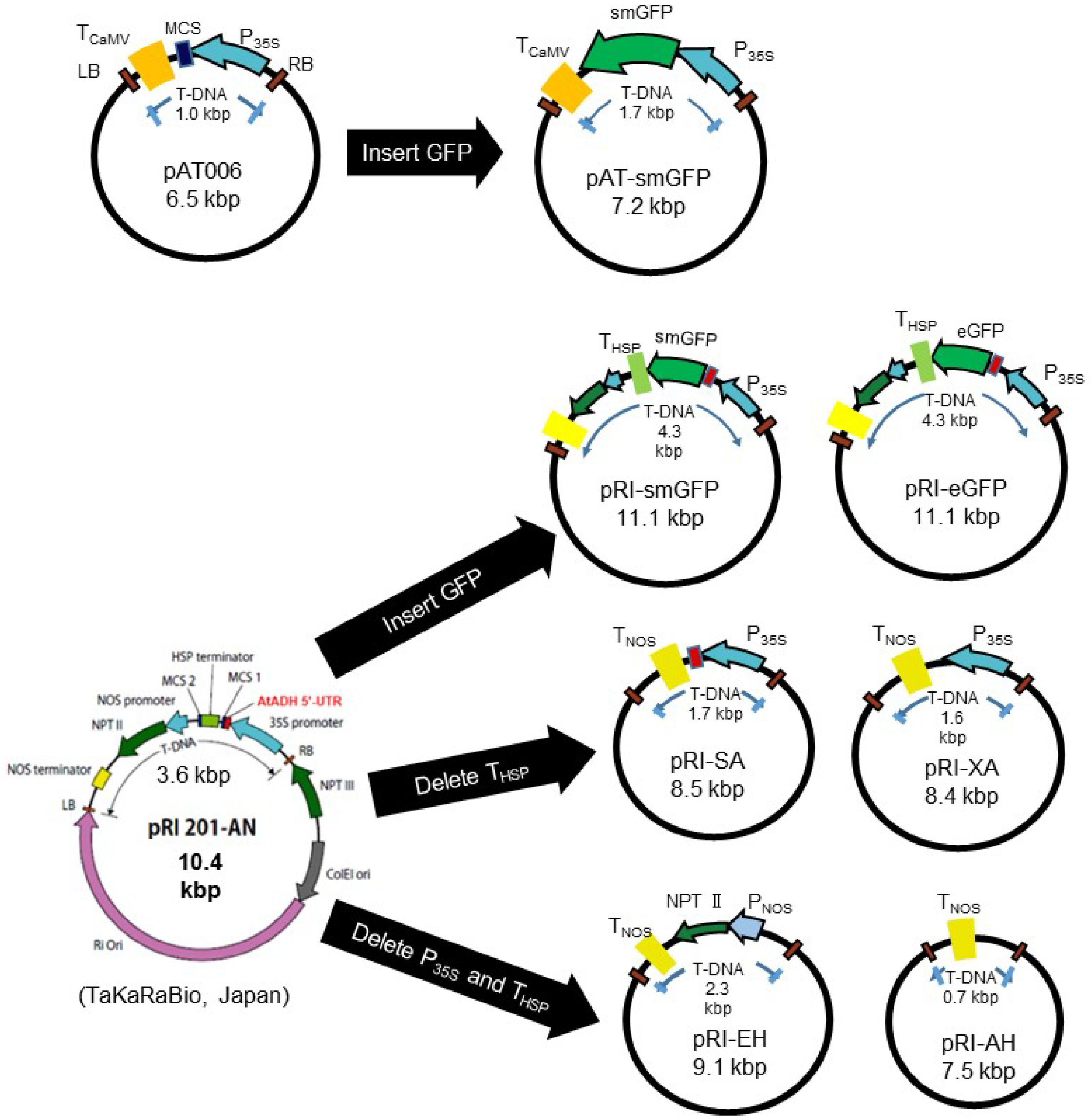

Supplementary Figure S1. Schematic drawings of T-DNA plasmids used in agroinfiltration. The binary plasmid pAT006 has a pMDC background (Tsuzuki et al., 2014), and pRI201-AN is commercially available from Takara Bio (Kusatsu, Japan). The construction procedure of their derivatives is described in the Materials and Methods. Abbreviations are as follows; P_{35S}, Cauliflower mosaic virus (CaMV) 35S promoter; P_{NOS}, Nopaline synthase (NOS) promoter; T_{CaMV}, CaMV terminator; T_{NOS}, NOS terminator; T_{HSP}, Heat shock protein (HSP) terminator derived from *A. thaliana;* eGFP, enhanced GFP; smGFP, soluble modified GFP; NPT, neomycin phosphotransferase; AtADH 5′-UTR, *A. thaliana* alcohol dehydrogenase 5’-untranslated region; RB, right border; LB, left border.

Tsuzuki M, Takeda A, Watanabe Y (2014) Recovery of dicer-like 1-late flowering phenotype by miR172 expressed by the noncanonical DCL4-dependent biogenesis pathway. RNA 20: 1320-1327

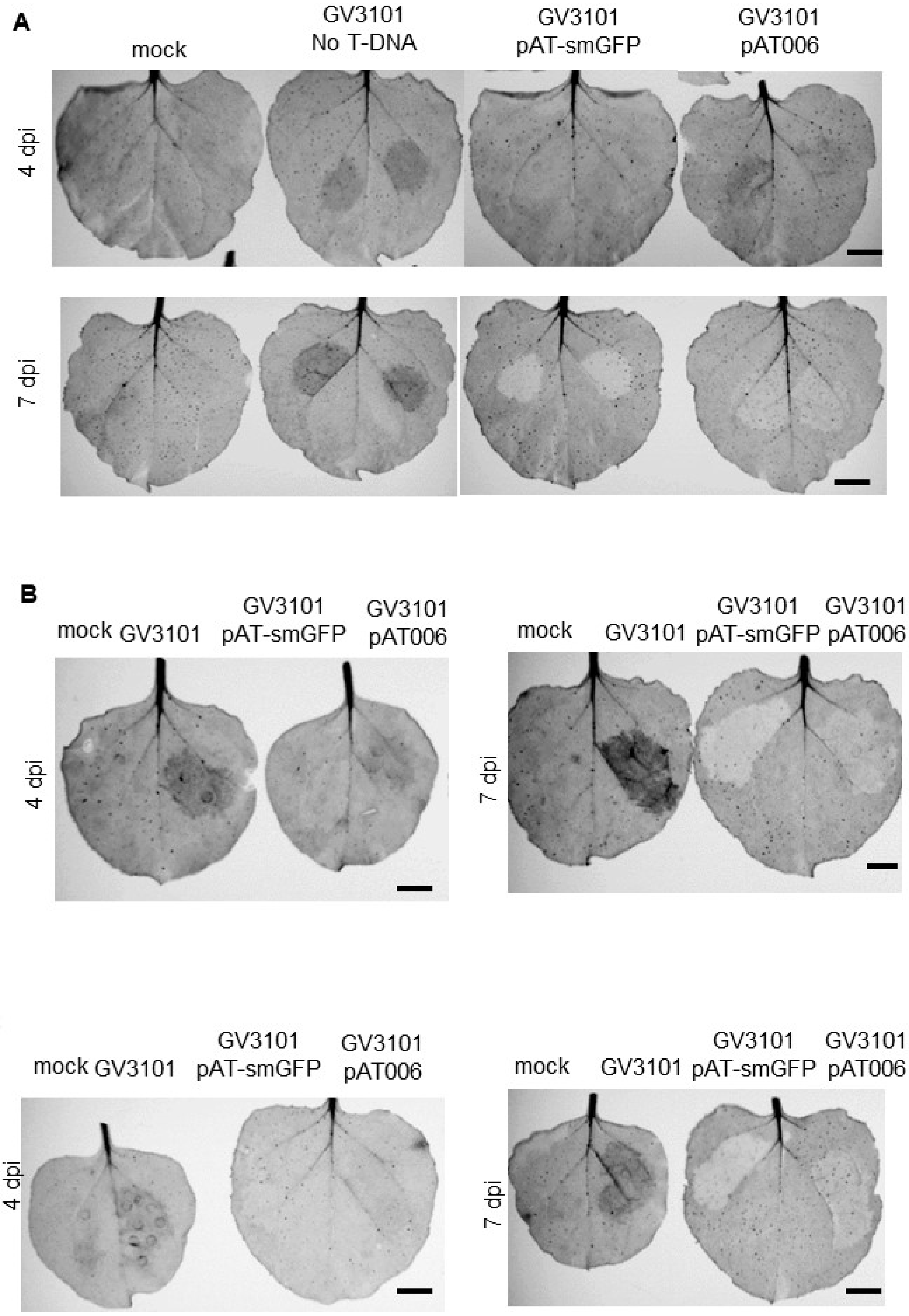
A
mock
GV3101
No T-DNA
GV3101
pAT-smGFP
GV3101
pAT006
4 dpi
7 dpi
B
mock
GV3101
GV3101
pAT-smGFP
GV3101
pAT006
4 dpi
7 dpi
C
mock
GV3101
GV3101
pAT-smGFP
GV3101
pAT006
4 dpi
7 dpi

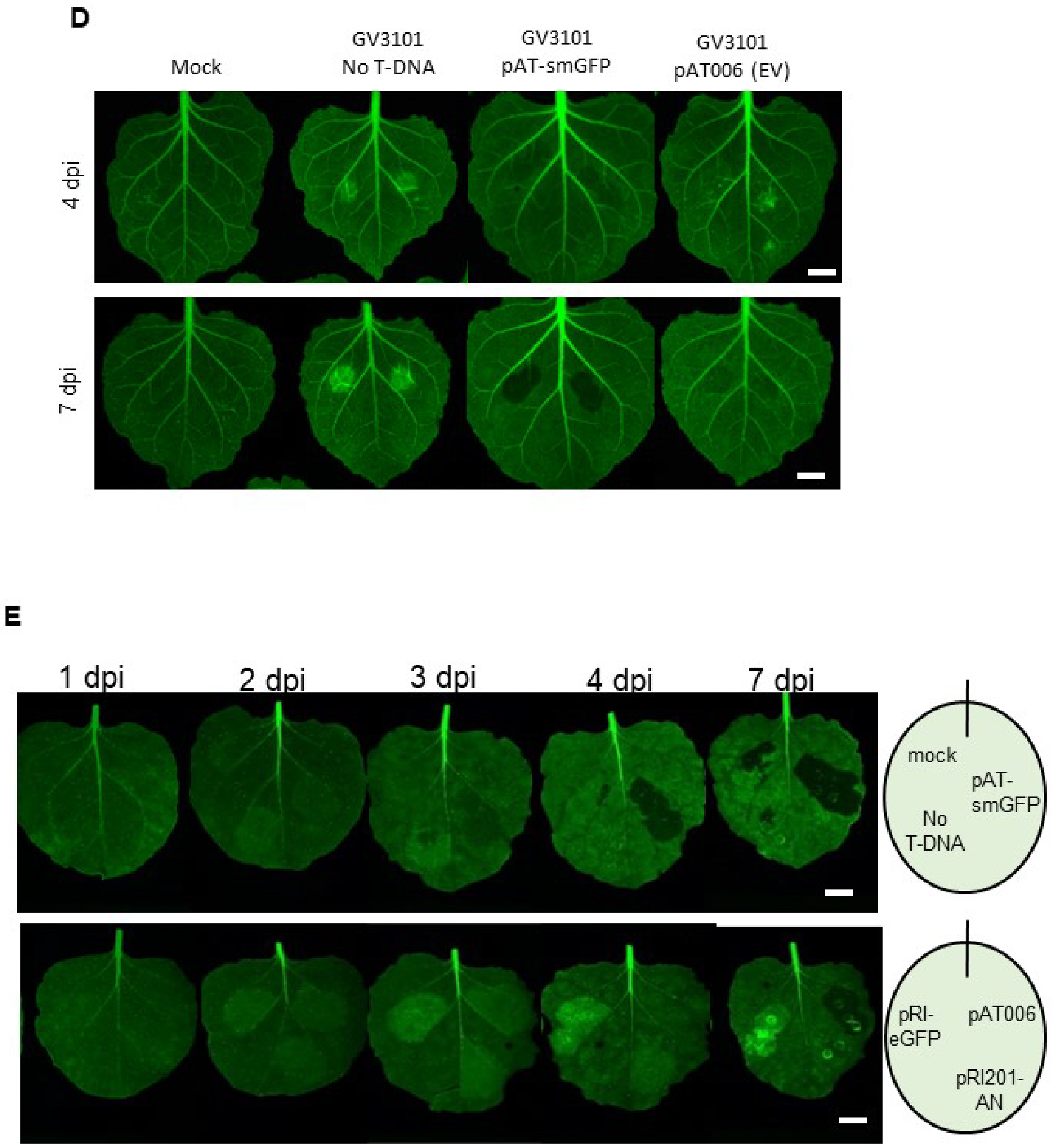

Supplementary Figure S2. Related to Figure 1. Induction of GFP gene silencing by infiltrating *A. tumefaciens* cells harboring the empty T-DNA plasmid as well as the GFP-expressing plasmid. Photographs of *N. benthamiana* leaves over-expressing the GFP gene (16c line), into which ag-robacteria were infiltrated, are shown. The results of five experiments are shown (A to E). Inten-sities of green fluorescence in black and white photographs and color photographs were measured by the imaging analyzer LAS-3000 (A - C; FUJIFILM, Japan) and the Fluorescence Imaging System, FOBI (D and E; NeoScience, Seoul, South Korea), respectively. Bars indicate 1 cm.

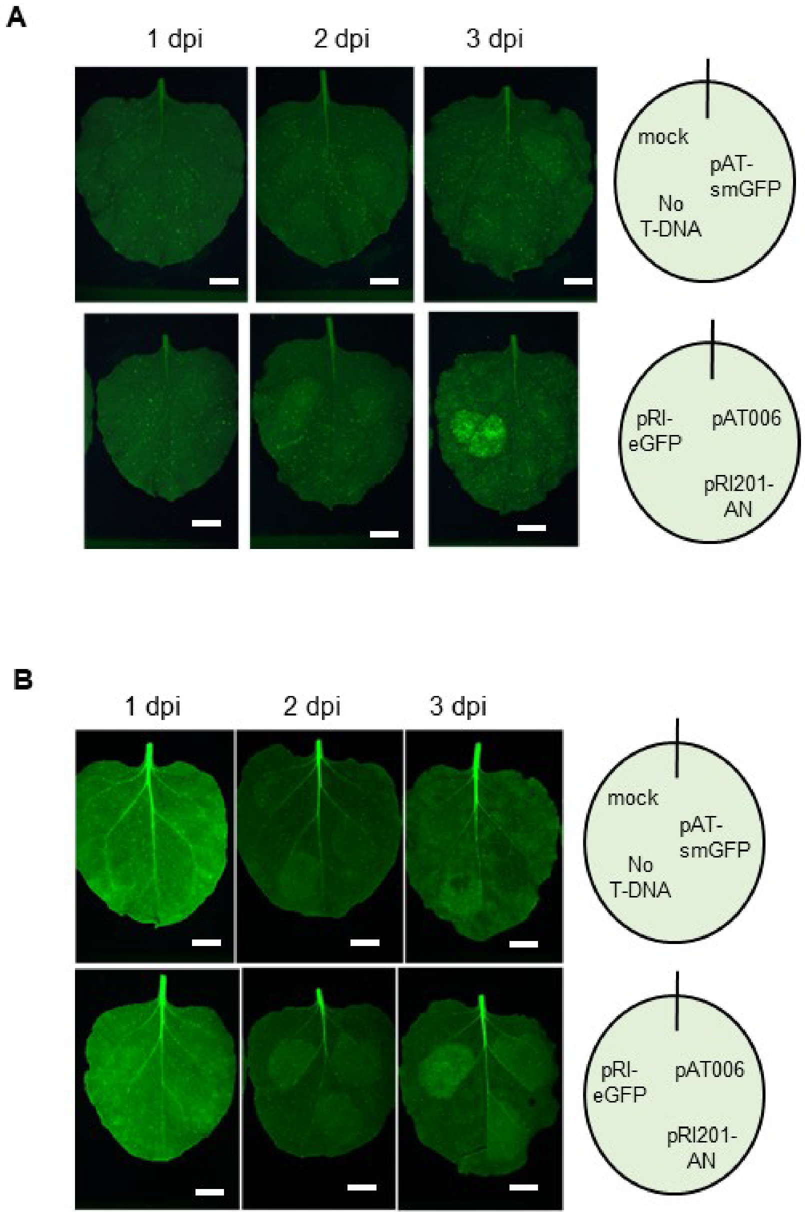

Supplementary Figure S3. Related to Figure 2. Induction of TGS by T-DNA insertion. Photographs of *N. benthamiana* leaves of WT (A) and 16c line (B), into which *A. tumefaciens* cells were infiltrated. Total RNA was isolated from the infiltrated sites of the *N. benthamiana* leaves shown here and Northern hybridization was performed. The results of Northern hybridization are shown in Figure 2. Bars indicate 1 cm.

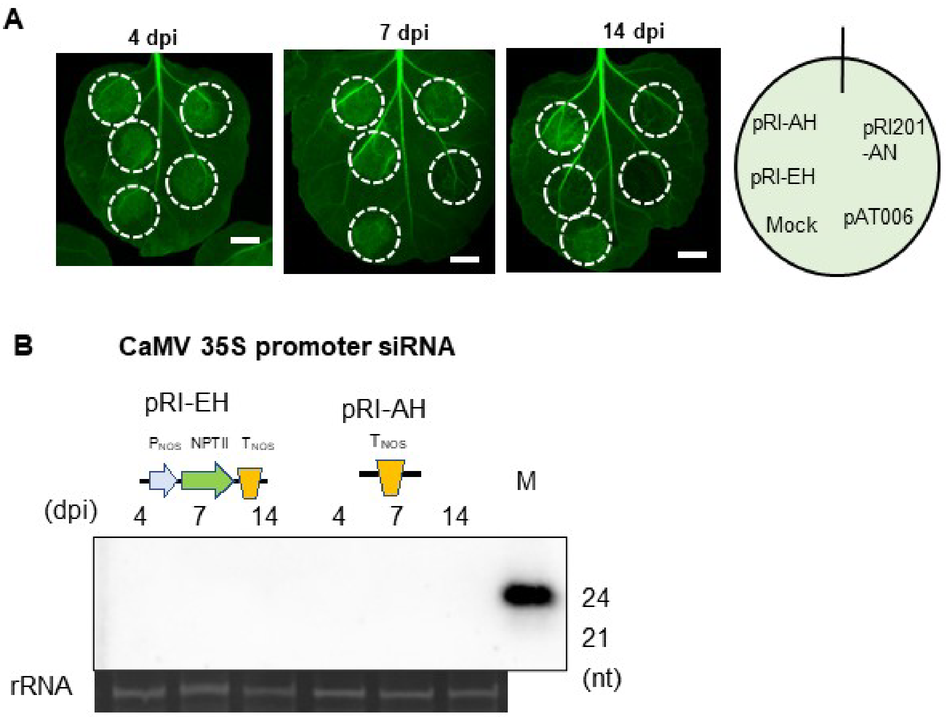

Supplementary Figure S4. Related to Figure 3. The structural feature of T-DNA to induce TGS. (A) Photographs of *N. benthamiana* leaves over-expressing the GFP gene (16c line), into which *A. tumefaciens* cells were infiltrated, are shown. Structures of plasmids are shown in Fig. S1. Green fluorescence was observed at 4, 7 and 14 dpi. White broken circles indicate infiltrated sites. (B) Detection of siRNAs derived from the CaMV 35S promoter by Northern hybridization. Total RNA was prepared from the infiltrated sites. Schematic drawings of T-DNA regions of two plasmids that were used in this experiment are shown. M indicates molecular weight marker of 24-nt ssRNA, and rRNA indicates rRNA bands stained by ethidium bromide as a loading control. Bars indicate 1 cm.

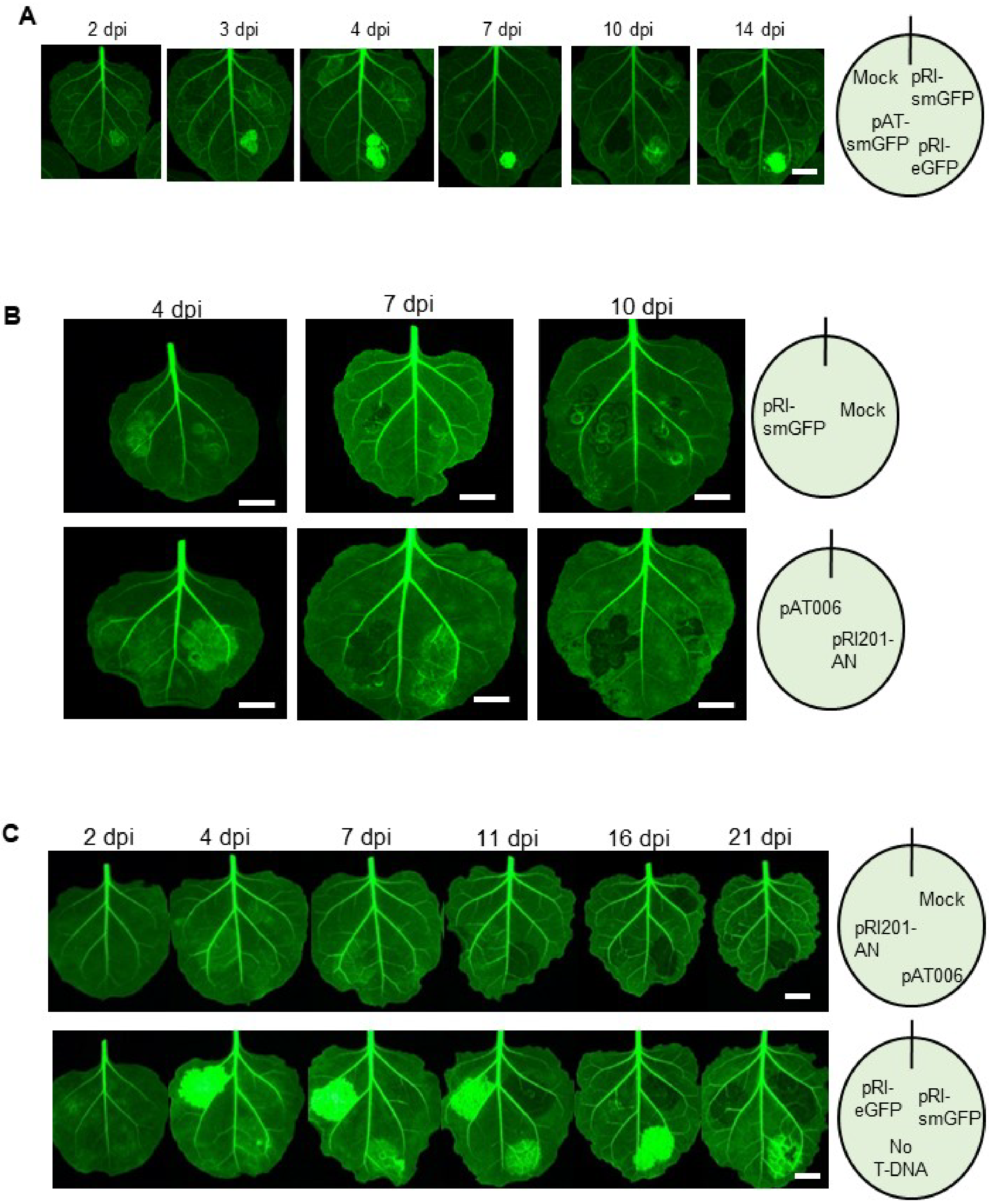

Supplementary Figure S5. Related to Figures 3 and 4. The structural feature of T-DNA to suppress the induction of TGS. Photographs of *N. benthamiana* leaves over-expressing the GFP gene (16c line), into which *A. tumefaciens* cells were infiltrated. The results of three experiments are shown (A) to (C). Total RNA was isolated from the infiltrated regions of the *N. benthamiana* leaves shown in (B) and (C), and Northern hybridization was performed. The results of Northern hybridization are shown in Figures 4B and 4C. Bars indicate 1 cm.

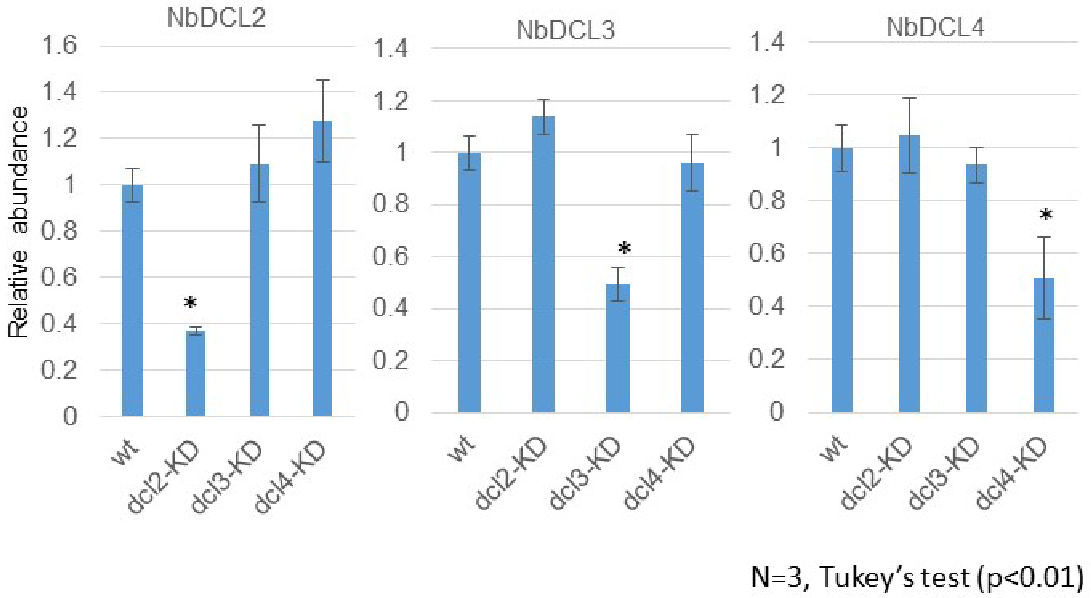

Supplementary Figure S6. Related to Figure 5. Confirmation of knocked-down of NbDCL genes in RNAi *N. benthamiana* plants by qPCR. Relative transcript abundance of three *N. benthamiana* Dicer-like protein genes (NbDCL2 to NbDCL4) in leaves was measured by qPCR. Data were obtained from three biological replicates. The actin gene (NbACT) was used as the internal standard. The amount of WT plants was set to 1 as the standard. Bars indicate ±SE, and significant differences (Tukey's test) are indicated by * ($p<0.01$).

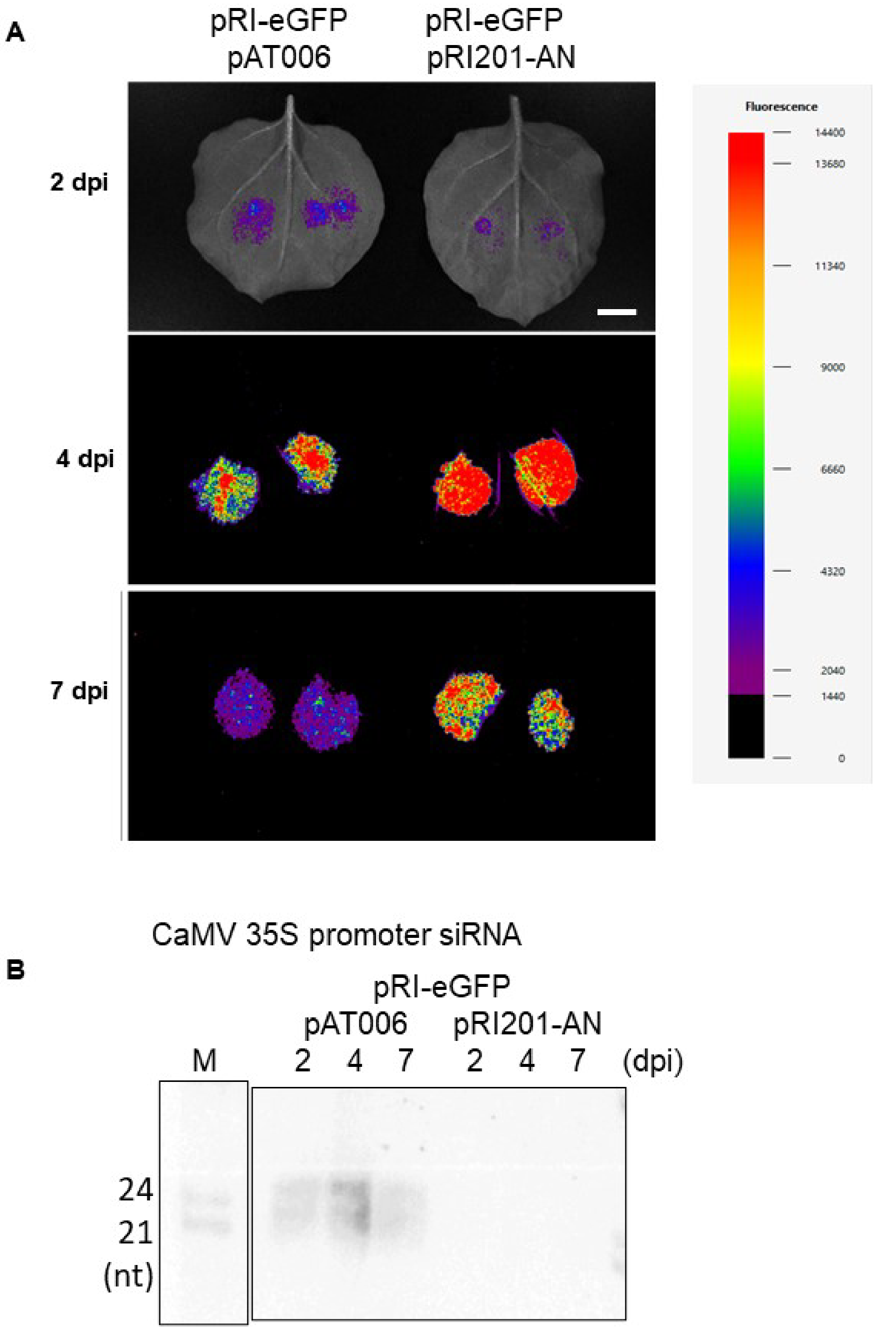

Supplementary Figure S7. Related to Figure 5. The induction of TGS to the GFP gene in a WT *N. benthamiana* plant by co-agroinfiltration. (A) Photographs of leaves of WT *N. benthamiana* plants, into which *A. tumefaciens* cells harboring T-DNA plasmids were co-infiltrated. *A. tumefaciens* cells harboring pRI-eGFP was mixed with either agrobacteria harboring pAT006 or those harboring pRI201-AN and then co-infiltrated into leaves of WT *N. benthamiana* plants. Green fluorescence was observed at 2, 4 and 7 dpi by the FOBI system and presented as pseudo-color by using the NEO image program. Bars indicate 1 cm. (B) Detection of siRNAs derived from the CaMV 35S promoter from co-infiltrated sites of agrobacteria harboring pRI-eGFP and those harboring pAT006 by Northern hybridization. M indicates molecular weight markers of 21-nt and 24-nt ssRNAs, and rRNA indicates 5S rRNA as a loading control.

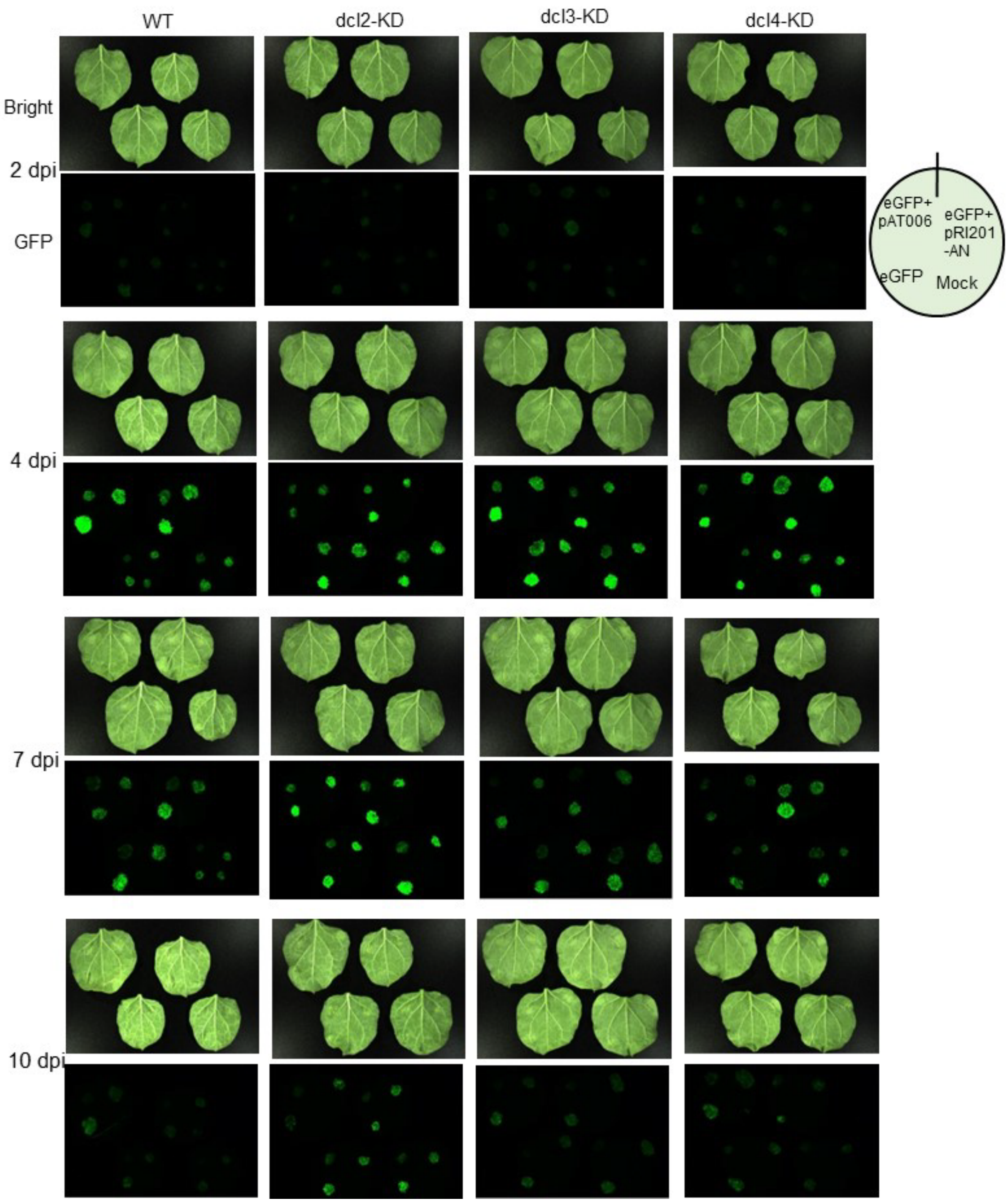

Supplementary Figure S8. Related to Figure 5. Involvement of siRNA-producing Dicers during TGS induction. Photographs of leaves of WT and three dcl-KD mutant plants of *N. benthamiana*, into which *A. tumefaciens* cells harboring T-DNA plasmids were co-infiltrated. *A. tumefaciens* cells harboring pRI-eGFP was mixed with either *A. tumefaciens* cells harboring pAT006 (GFP+pAT) or those harboring pRI201-AN (GFP+pRI), and then co-infiltrated into leaves of WT and three dcl-KD mutant plants of *N. benthamiana*. Only *A. tumefaciens* cells harboring pRI-eGFP was also infiltrated as a control (GFP), and Mock indicates mock inoculation with agroinfiltration buffer. Green fluorescence was observed at 2, 4, 7 and 10 dpi by the FOBI system. Intensities of green fluorescence in agroinfiltrated sites were quantified and analyzed by the NEO image program and shown in Figure 5B and Supplementary Figure S7.

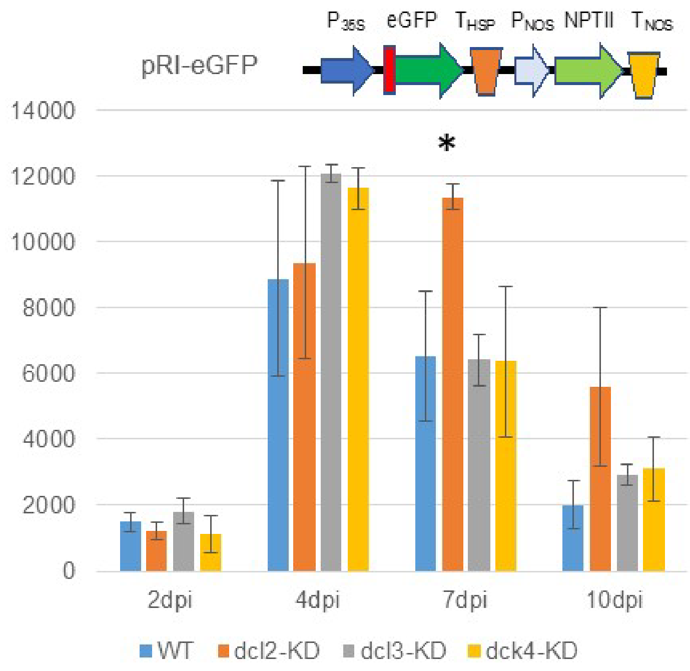

Supplementary Figure S9. Related to Figure 5. Involvement of siRNA-producing Dicers during TGS induction. *A. tumefaciens* cells harboring T-DNA plasmids were co-infiltrated into leaves of WT and three dcl-KD mutant plants of *N. benthamiana*. Photographs of leaves infiltrated with agrobacteria harboring pRI-eGFP+pAT006, pRI-eGFP+pRI201-AN, pRI-eGFP and Mock are shown in Figure 5 and Supplementary Figure S7. Intensities of green fluorescence in infiltrated sites were quantified and analyzed by the NEO image program. Bars indicate ±standard errors (SE) of four biological replicates, and significant differences (Tukey's test) are indicated by * ($p<0.01$). Results of pRI-eGFP+pAT006 and pRI-eGFP+pRI201-AN) are shown in Figure 5B, and the results of pRI-eGFP are shown here.
